# Factors affecting providers’ delivery of intermittent preventive treatment for malaria in pregnancy: a five-country analysis of national service provision assessment surveys

**DOI:** 10.1186/1475-2875-13-440

**Published:** 2014-11-20

**Authors:** Mathieu Maheu-Giroux, Marcia C Castro

**Affiliations:** Department of Global Health & Population, Harvard School of Public Health, 665 Huntington Avenue, Bldg I, Room 1113, Boston, MA 02115 USA

## Abstract

**Background:**

Intermittent preventive treatment in pregnancy (IPTp) delivered during antenatal care (ANC) visits has been shown to be a highly efficacious and cost-effective intervention. Given the high rates of ANC attendance in sub-Saharan Africa, the current low IPTp coverage represents considerable missed opportunities. The objective of this study was to explore factors affecting provider’s delivery of IPTp during ANC consultations.

**Methods:**

Data from five nationally representative service provision assessment surveys informed the statistical analyses (Kenya, Namibia, Rwanda, Tanzania, and Uganda; 2006-2010). Poisson regression models with robust/clustered standard errors were used to estimate the effect of different determinants on IPTp delivery from 4,971 observed ANC consultations.

**Results:**

The five major modifiable determinants of IPTp delivery were: 1) user-fees for ANC medicines (relative risk (RR) = 0.76; 95% confidence intervals (95% CI): 0.62-0.93); 2) facilities having IPTp guidelines (RR = 1.12; 95% CI: 1.01-1.24); 3) facilities having implemented IPTp as part of their routine ANC services offering (RR = 4.18; 95% CI: 1.75-10.01); 4) stock-outs of sulphadoxine-pyrimethamine (RR = 0.40; 95% CI: 0.27-0.60); and, 5) providers having received IPTp training (RR = 1.21; 95% CI: 1.09-1.35). Using the population-attributable fraction, it was estimated that addressing these barriers jointly could lead to a 31% increase in delivery of this intervention during ANC consultations. Of these four potentially modifiable determinants, training of providers for IPTp had the largest potential impact.

**Conclusions:**

If proved to be cost-effective, dispensing IPTp training to ANC providers should be prioritized. Multifaceted approaches targeted in areas of low coverage and/or type of facilities least likely to provide this intervention should be implemented if the Roll Back Malaria target of 100% IPTp coverage by 2015 is to be attained.

**Electronic supplementary material:**

The online version of this article (doi:10.1186/1475-2875-13-440) contains supplementary material, which is available to authorized users.

## Background

Pregnant women are especially vulnerable to malaria as parasitaemia during pregnancy can lead to serious adverse maternal, foetal, and infant health outcomes [1]. In sub-Saharan Africa, where malaria burden is concentrated, there was an estimated 30 million pregnancies at risk of this parasitic disease in 2007 [2]. The World Health Organization (WHO) recommends a three-pronged approach to address this major public health issue: 1) prompt and effective case management of clinical malaria and anaemia; 2) distribution of insecticide-treated bed nets; and, 3) intermittent preventive treatment in pregnancy (IPTp) with sulphadoxine-pyrimethamine (SP) [3]. IPTp consists of presumptive provision of anti-malarials to pregnant women, shortly after quickening and at intervals of at least four weeks, under the direct observation of health workers. This intervention has been shown to be safe and highly effective to prevent maternal anaemia, low birth weight, and neonatal mortality [4–7], even in areas of recorded resistance to SP [8]. Further, IPTp delivered through antenatal care clinics (ANC) is considered very cost-effective with an incremental cost-effectiveness ratio of $1.02 (2007 USD) per disability-adjusted life-year averted [9].

Despite the fact that 75% of pregnant women in sub-Saharan Africa visit ANC services at least twice during their pregnancy, the proportion of women receiving at least two SP doses remained stubbornly low at 22% in 2009-2011 [10, 11]. Such missed opportunities to deliver IPTp through ANC have been described as a ‘*failure of the public health community*’ [12]. Further, the actual coverage levels of IPTp are disconcerting given the Roll Back Malaria Partnership goal of 80% IPTp coverage with at least two SP doses by 2010 (and 100% by 2015) [13] and the President’s Malaria Initiative’s target of 85% coverage [14].

A systematic review of qualitative studies that explored barriers to IPTp coverage showed that unclear policy guidance, drug stock-outs, user fees, poor organization at health facility, underperformance of healthcare providers, and low ANC attendance were effectively impeding delivery of this intervention [12]. Other authors have also suggested that confusion among healthcare providers, related to the previous WHO guidelines regarding timing and number of IPTp doses, was one of the key factors influencing coverage of this intervention [11, 15]. These recommendations were recently revised in an attempt to provide clearer guidance and WHO now advocates that SP should be provided at each of the four scheduled antenatal care visits [16]. A recent meta-analysis found that the number and timing of ANC visits, parity, education level, socio-economic status, knowledge about malaria and IPTp, and use of ITN were key determinants of IPTp uptake at the individual level [12]. Few interventions have so far been proposed to increase coverage of IPTp. Community-based distribution could effectively complement ANC-based delivery of IPTp [17, 18] but there is some evidence that this could concurrently reduce women’s ANC attendance [19, 20]. Potential alternatives were principally aimed at promoting IPTp in the community, increasing ANC coverage, and improving providers’ performance [21–23].

In light of the important gap between ANC attendance and IPTp uptake, it is hypothesized that interventions aimed at health providers are likely to be most cost-effective. A multi-country quantitative analysis of factors affecting providers’ delivery of IPTp has yet to be conducted, however. The aim of this study is to investigate providers’ determinants of IPTp delivery in sub-Saharan Africa. Such information could provide crucial evidence to effectively design and implement interventions leading to significant increases in IPTp coverage in malaria-endemic areas. To this end, service provision assessment (SPA) surveys, conducted in five countries where a national IPTp policy was implemented at the time of the survey (Kenya, Namibia, Rwanda, Tanzania, Uganda), were used. By comprehensively assessing the preparedness of health facilities to provide maternal care and collecting detailed and standardized information on the performance of IPTp delivery systems, these nationally representative surveys provide a unique opportunity to explore supply-side determinants of IPTp delivery.

## Methods

SPA surveys have been conducted by The DHS Program since 1999 in 11 countries so far. Standardized methodology and instruments are used to provide comparable information on key indicators of formal sector health services related to child health, maternal and newborn health, family planning, selected infectious diseases services, basic surgery, and non-communicable diseases. As such, pharmacies and individual doctor’s practices are not usually included in these surveys. SPA surveys consist of four main questionnaires: 1) an inventory questionnaire that collects information on availability of different services and general service readiness at the health facility; 2) a health worker interview questionnaire; 3) an observation protocol of client-provider consultations for selected services; and, 4) an exit interview questionnaire for clients of these selected services.

### Inclusion criteria

Only recent surveys conducted after 2000, that implemented the observation protocol for ANC client-provider consultations, and that were performed at a time when the country had implemented a national IPTp policy were included. For this reason the SPAs from Namibia and Rwanda are included in this study even though they abandoned their national IPTp policy in 2010 and 2008, respectively, following important declines in malaria transmission experienced by these two countries. Hence, data on delivery of IPTp and its determinants came from five SPA surveys conducted between 2006 and 2010 in Kenya (2010) [24], Namibia (2009) [25], Rwanda (2007) [26], Tanzania (2006) [27], and Uganda (2007) [28]. The Kenya 2004 SPA [29] was not included because interviewers did not record whether anti-malarial prophylaxis was observed to be administered as directly observed therapy (DOT) or only prescribed to the ANC clients.

### Survey design

The sampling for each SPA was designed to allow for indicators to be representative at the national and regional levels, by type of facility and by managing authority. The sample of facilities selected for inclusion was obtained from a master list of all health facilities. National referral hospitals and regional general hospitals are often oversampled, as well as facilities providing specific services. Survey weights are assigned to each facility to correct for such differential sampling. Three of the included SPA surveys used this design. In contrast, Namibia performed a census of all its health facilities, and Rwanda included all of its government-owned establishments, all private facilities with at least five employees, and one third of private facilities with three or four employees.

Providers of health services were sampled among those who were present in the facility on the day of the survey and who provided the services being assessed by the SPA survey. The target was to interview an average of eight providers by facility, including all those whose consultations were observed. Sampling weights were constructed in order to account for differential sampling of providers with distinct qualifications in a facility type and region. The sample of health providers was generally considered representative of the staff who provides the services being assessed, except in the very few instances when a special training event or evaluation for a group of workers would have taken them away from their post on the day of the survey.

It is generally difficult to obtain a sampling frame of eligible clients that would attend the assessed services on any particular day. Therefore, the SPA surveys used a convenience sample of clients for the observation protocols and exit interviews. Specifically, clients were selected as they arrived for consultations at the facility. When multiple eligible clients were available, interviewers selected two new clients for every follow-up case. A target of five clients was included, with a maximum of 15 observations in any given facility for each assessed service. Exit interviews were attempted for every client observed during a consultation. All SPA surveys used this sampling design except the 2010 Kenyan SPA. For this latter survey, clients entering the facility were systematically sampled until a maximum of five observations per provider were obtained and no more than 15 observations in any given facility for each service. Sampling weights used the facility weights described above and adjusted for over-representation of observations based on the compiled total number of clients of each service of interest seen on the day of the survey (note that facility, providers and clients’ sampling weights are not provided in the Rwanda 2007 SPA). In a few instances, the sample of clients present on the day of the survey might not be representative of clients normally receiving health services if the survey coincided with special events such as a health fair or campaign.

### Data processing

Databases with observations of ANC consultations and exit interviews were linked to the provider’s interview and facility questionnaires using their unique identifiers. Although the survey instruments described above were standardized, country differences in the classification of the different facility types and of a provider’s qualifications exist. Hence, the classification of these variables was harmonized according to the criteria described in Additional file 1 before merging the five SPAs together. The variables considered in this study were:

**Facility level:** 1) facility type; 2) managing authority (public *vs* private); 3) whether the facility charged user-fees for medicines given during ANC consultations (yes/no, as reported by the manager of ANC services); 4) whether the facility had guidelines or protocol for IPTp; 5) whether the manager of ANC services claimed that IPTp was routinely offered to antenatal clients; and, 6) whether the facility had tablets of SP (Fansidar, Metakelfin, Orodar) available in its inventory on the day of the survey (stock-outs are defined as not having SP in the inventory on the day of the survey). Information on these variables was extracted from the facility questionnaire.

**Provider level:** 1) professional/technical/medical qualification of the provider; 2) whether the provider reported to have received supervision or technical support from a supervisor in the facility or outside of the facility in the previous six months; and, 3) whether the provider had received any pre-service or in-service training for IPTp in the preceding year. Information on these variables was extracted from the providers’ questionnaire.

**Client level:** 1) whether the provider administered anti-malarial prophylaxis as DOT (as observed by the interviewer); 2) primigravidae status; 3) whether it was the first ANC visit at the facility for the current pregnancy; 4) education level; 5) age; and, 6) length of pregnancy (weeks). Information on these variables was extracted from the observation protocol of client-provider consultations and the clients’ exit interview questionnaire.

Namibia’s IPTp policy only targeted its malaria-endemic areas and observations of consultations outside these areas were therefore excluded. Similarly, the policy in place in Kenya during the 2010 SPA specifically targeted three provinces where malaria was most endemic (Nyanza, Coast, and Western) [24] but the decision to include observations from all provinces was made because IPTp delivery did not differ between the target and non-targeted provinces (i.e., 44 *vs* 42%). Finally, because IPTp administration should be avoided in the first trimester (i.e., before quickening), only consultations from clients that were 16 weeks pregnant or more were included.

### Statistical analyses

The main outcome is whether the provider was observed to provide anti-malarial prophylaxis as DOT during an ANC consultation (hereinafter referred to as IPTp). The frequency of this outcome was high and reporting odds ratio will overstate the relative risk (RR) association - the quantity of interest for public health research. Log-Poisson models provide consistent estimates of RR but, in case of common binary outcomes, Poisson errors will overestimate binomial errors if a robust error variance is not used [30]. Hence, a modified Poisson regression model that used generalized estimating equation to perform the unbiased variance estimation, taking into account clustering of observations within providers and facilities [31, 32], was adopted. This model takes the following form:


where π_*ijk*_ is the probability that women *i* is being given anti-malarial prophylaxis by provider *j* in facility *k*; *α* is the intercept; *β* is a vector of coefficients for facility-level variables; *δ* is a vector of coefficients for provider-level variables; *γ* is a vector of coefficients for the individual women’s variables; and *ω* is the vector of coefficients for country fixed effects. Robust standard errors were obtained using an exchangeable correlation structure at the facility level. Because observations of client-provider’s consultation are perfectly nested within facilities, clustering the standard errors at this upper level through the sandwich variance estimator will also take into account clustering of consultations at the provider level [33]. Following the rationale of Solon *et al*. [34], who discussed three situations where using sampling weights is justified (none of which applied to the present case), sampling weights were not used in the regression analyses.

Exploratory data analyses suggested that the relationship between the outcome and week of pregnancy was not linear. The functional form of this variable was hence modelled using a cubic b-spline with five degrees of freedom. Observations with missing values for the outcome were excluded from the analysis and covariates with missing observations were retained in the analysis using the missing indicator method [35]. All variables were first entered in univariate models before the full multivariate model was fit. Pooling data from these five countries was deemed appropriate as key barriers to delivery of IPTp have been shown to be relatively consistent across countries [12]. Further, preliminary analyses of separate regression models for each country, where confidence intervals of the estimates were overlapping, suggest that there was no significant effect modification by country (Additional file 2). All analyses were performed using the R statistical software [36], and the ‘*geepack*’ package [37] was used to fit the modified Poisson regression models with clustered standard errors.

In order to quantify the contribution of selected potentially modifiable determinants of IPTp delivery (defined as the provision of any dose of IPTp-DOT), the population attributable fraction (PAF) was estimated. The PAF quantifies the proportional increase in IPTp delivery during ANC consultations that would have occurred if a barrier to delivery had been completely removed or a driver fully scaled-up. The following equation was used to compute this metric:


where *RR* is the relative risk for the selected determinant; *P*_*0*_ is the proportion of the currently non-exposed population; and *P*_*1*_ is the proportion of the currently exposed population. The exposed and unexposed proportions of the population were calculated using the appropriate sampling weights. These weights were multiplied by the number of reported ANC visits per month for each facility to obtain estimates representative of all women seeking ANC services. The five surveys were combined and each survey was re-weighted proportionally to the size of its country's female population. For cases where the determinant was a barrier (i.e., RR < 1), the coding of the exposure was reversed so that the reference exposure (non-exposed) is changed to the exposed and the inverse of the RR was inputted in the modified PAF formula. Uncertainty intervals (UI) for this modified version of the PAF were obtained using 10,000 Monte Carlo simulations.

Finally, the joint contribution of multiple modifiable determinants of IPTp delivery, taking into account their potential correlation, was assessed. Because preliminary analyses have shown that the selected determinants do not interact on the multiplicative scale, their joint contribution was estimated using a multiplicative excess risk scale. The joint PAF for multiple determinants was computed by summing the combined RR of individual records using the appropriate survey weights [38]. This joint PAF was calculated using the following formula:


where *β*_*j*_ corresponds to the log RR per unit of exposure of determinants *j*; *X*_*ij*_*’* is a vector that contains the alternative distribution of determinants *j* for each record *i* (for this study, binary determinants were set to 1, corresponding to the situation were all providers would be exposed); and *X*_*ij*_ is the current distribution of determinant *j* for each record *i*. As for the individual PAF, the coding of barriers (i.e., RR < 1) was reversed and the inverse of the RR was used. To calculate uncertainty intervals for the joint PAF, 10,000 log-RR from a multivariate normal distribution were first simulated. These Monte Carlo simulations were then combined with 10,000 bootstrap replicates of the current exposure distribution, implicitly modelling the correlation structure of the different exposures and defining the sampling unit at the facility level to take into account clustering of observations. These analyses were also performed using the R statistical software [36].

## Results

**Figure 1:**
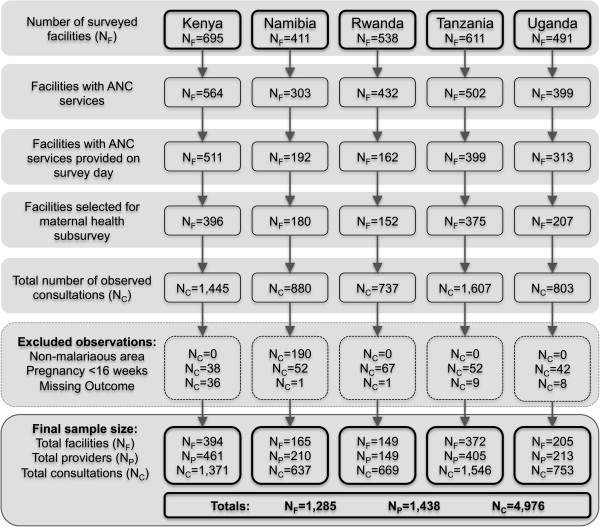
**Flowchart of the inclusion of the health facilities, providers, and clients of ANC services in the service provision assessments survey of Kenya, Namibia, Rwanda, Tanzania, and Uganda (2006-2010).**

A total of 2,746 health facilities were surveyed in the five combined SPA surveys, of which 2,200 provided ANC services (Figure 1). Among these, 1,577 were offering this type of services on the day of the survey and 1,310 health facilities were further selected for the maternal health subsurvey with observations of client-provider’s ANC consultation and client exit interviews. After excluding observations of consultations performed in non-malarious areas of Namibia (n = 190), those for women less than 16 weeks into their pregnancy (n = 251), and those for which the main IPTp outcome was missing (n = 55), a total of 4,976 observations of client-provider consultations contributed information to the analyses. These consultations were performed in 1,285 different facilities by 1,438 unique providers. The great majority of facilities (89.9%) had only one provider contributing information and each provider had an average of 3.5 observed ANC consultations.

The characteristics of facilities, providers and clients are described in Table 1. Anti-malarial prophylaxis was administered as DOT in only 35% of all consultations. There were important variations in the proportion of different types of facilities surveyed between countries, reflecting national differences in health system delivery of ANC services. For example, 94.6% of facilities providing ANC services in Rwanda were health centres, but this proportion dropped to 8.3% in Tanzania. Namibia’s health system is almost entirely public and less than 1% of facilities in malarious areas of this country charged user-fees for ANC medicines. In contrast, private practices and user fees were more common in Kenya. Overall, 93% of the surveyed facilities claimed to routinely provide IPTp as part of their ANC services but this proportion varied from a low of 73% in Namibia to a high of 99% in Uganda. SP was not available in 11% of the facilities on the day of the survey. The great majority of providers of ANC services were either enrolled nurse/midwife (36%) or registered nurse/midwife (42%), and only 26% of all providers had received IPTp training in the year preceding the survey.

**Figure 2:**
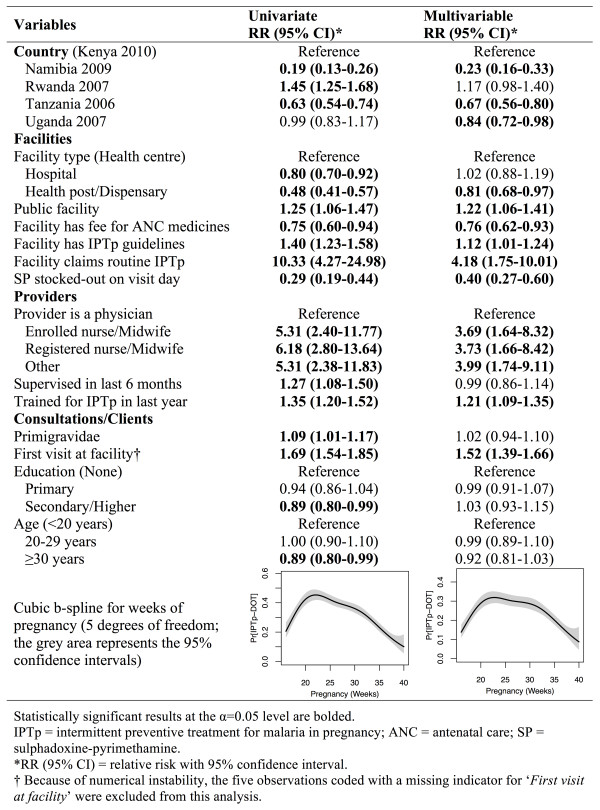
**Univariate and multivariable results of the modified Poisson regression model of providers’ determinants of delivery of intermittent preventive treatment for malaria in pregnancy administered as directly observed therapy (N = 4,971).**

Results from univariate and multivariable regressions (Figure 2) show that hospitals and health centres did not differ much in their propensity to deliver IPTp but health posts and dispensary were 20% less likely to deliver IPTp as compared to health centres. Providers working in public facilities are, however, 22% more likely to provide anti-malarial prophylaxis to their ANC clients. Importantly, user-fees for ANC medicines seem to deter client from receiving IPTp in both univariate and multivariable analyses with an adjusted RR of 0.76 (95% confidence interval (95% CI): 0.62-0.93). Having IPTp guidelines or protocols in the facilities was also associated with increased delivery of this intervention (RR = 1.12; 95% CI: 1.01-1.24). Unsurprisingly, the factor whose effect was greatest was whether the facility claimed that IPTp was routinely offered as part of their ANC services. Such facilities were close to four times more likely to deliver this intervention. An important barrier to IPTp was stock-outs of SP on the day of the survey. Consultations occurring in stocked-out facilities were 60% less likely (RR = 0.40; 95% CI: 0.27-0.60) to deliver IPTp. Even if SP was stocked-out in the facility’s inventory on the day of the survey, providers could have administered IPTp if a separate supply was kept in the office were ANC consultations occurred, explaining why some women still received IPTp in stocked-out facilities.

**Table 1:** **Characteristics of facilities, providers and of the observed women’s antenatal care consultations (if ≥16 weeks pregnant) for five service provision assessment surveys conducted in sub-Saharan Africa**

Variables	Kenya 2010	Namibia 2009	Rwanda 2007	Tanzania 2006	Uganda 2007	All combined
**Facility**	**N = 394**	**N = 165**	**N = 149**	**N = 372**	**N = 205**	**N = 1,285**
Facility type						
Health centre	25.6%	19.4%	94.6%	8.3%	27.3%	28.1%
Hospital	52.0%	2.4%	2.7%	30.1%	41.0%	31.8%
Health post/dispensary	22.3%	78.2%	2.7%	61.6%	31.7%	40.1%
Public facility	67.3%	99.4%	70.5%	80.4%	77.1%	77.1%
Facility has fee for medicines	31.7%	0.6%	4.7%	4.3%	3.9%	12.2%
*Missing*	*0.5%*	*0.0%*	*5.4%*	*0.0%*	*0.5%*	*0.9%*
Facility has IPTp guidelines	53.0%	3.0%	50.3%	51.3%	47.8%	45.0%
Facility claims routine IPTp	95.2%	72.7%	96.0%	94.6%	99.5%	92.9%
*Missing*	*0.0%*	*0.0%*	*0.0%*	*0.5%*	*0.0%*	*0.2%*
SP stocked-out on visit day	8.6%	22.4%	10.7%	12.1%	6.8%	11.4%
*Missing*	*0.8%*	*0.6%*	*0.0%*	*0.8%*	*0.0%*	*0.5%*
**Provider**	**N = 461**	**N = 210**	**N = 149**	**N = 405**	**N = 213**	**N = 1,438**
Type of provider						
Physician	5.9%	0.0%	2.0%	5.7%	3.3%	4.2%
Enrolled nurse/midwife	47.5%	59.5%	2.7%	14.1%	50.2%	35.6%
Registered nurse/midwife	42.7%	38.6%	79.2%	34.6%	33.8%	42.3%
Other	2.4%	1.0%	15.4%	44.4%	7.0%	16.1%
*Missing*	*1.5%*	*1.0%*	*0.7%*	*1.2%*	*5.6%*	*1.9%*
Supervised in last 6 months	78.3%	70.5%	86.6%	78.8%	79.3%	78.3%
*Missing*	*1.7%*	*1.0%*	*1.3%*	*1.2%*	*6.1%*	*2.1%*
Trained for IPTp in last year	31.2%	25.7%	30.2%	16.5%	32.4%	26.3%
*Missing*	*2.2%*	*1.0%*	*0.7%*	*1.2%*	*6.1%*	*2.2%*
**Consultations/clients**	**N = 1,371**	**N = 637**	**N = 669**	**N = 1,546**	**N = 753**	**N = 4,976**
IPTp administered as DOT	41.8%	8.0%	59.0%	25.4%	42.0%	34.7%
Prescribed/Given IPTp	67.5%	19.1%	69.3%	49.1%	65.2%	55.4%
*Missing*	*0.2%*	*0.3%*	*1.0%*	*0.1%*	*0.5%*	*0.3%*
Primigravidae	31.1%	35.2%	28.7%	24.3%	30.5%	29.1%
*Missing*	*1.0%*	*0.2%*	*0.6%*	*0.1%*	*0.3%*	*0.4%*
First visit at facility	38.9%	50.7%	50.2%	39.4%	46.6%	43.2%
*Missing*	*0.1%*	*0.0%*	*0.0%*	*0.0%*	*0.0%*	*0.0%*
Education						
None	8.2%	11.9%	35.1%	21.9%	19.8%	18.3%
Primary	49.9%	25.0%	54.9%	66.7%	48.5%	52.4%
Secondary/Higher	39.2%	63.1%	7.8%	11.3%	29.0%	27.8%
*Missing*	*2.7%*	*0.0%*	*2.2%*	*0.1%*	*2.8%*	*1.5%*
Age						
<20 years	13.7%	21.5%	4.0%	15.5%	21.1%	15.1%
20-29 years	63.4%	49.5%	59.2%	57.8%	56.8%	58.3%
≥30 years	19.9%	25.7%	34.4%	26.6%	19.1%	24.6%
*Missing*	*3.0%*	*3.3%*	*2.4%*	*0.2%*	*2.9%*	*2.1%*
Weeks of pregnancy (mean)	29.9	27.9	26.5	27.9	28.8	28.4

Descriptive statistics do not take into account survey weights.

ANC = antenatal care; IPTp = intermittent preventive treatment for malaria in pregnancy; DOT = directly observed therapy.

At the provider level, clinicians were the least likely to deliver the IPTp intervention. Providers with other qualifications did not exhibit much difference in their estimated effect size measures. Providers who had been supervised in the previous six months were not more likely to deliver IPTp during a consultation. In contrast, providers who have received IPTp training during the previous year were 21% more likely to give anti-malarial prophylaxis during ANC visits (RR: 1.21; 95% CI: 1.09-1.35). Providers whose clients were seeking ANC services for the first time in their current pregnancy were 52% (RR = 1.52; 95% CI: 1.39-1.66) more likely to give them IPTp while age of the client was not statistically significant in the multivariable analysis. Finally, clients in their 20^th^ to 32^nd^ week of pregnancy were the most likely to receive IPTp.

All these results were robust to different model specifications. Specifically, the different effect size measures were robust to the inclusion of 60 dummy variables representing the different subnational regions (Additional file 3). Including fixed effects at the regional level could effectively account for unmeasured differences in malaria endemicity levels or regional targeting by national malaria control programmes. Further, restricting the analyses to consultations from providers working in facilities where IPTp was claimed to be routinely offered as part of their ANC services had no impact on the other determinants’ point estimates (Additional file 4). Finally, when the outcome was defined as providers prescribing or giving anti-malarial prophylaxis – without considering if it was administered as DOT – all determinants remained statistically significant (except for the availability of IPTp guidelines/protocols), although the point estimates were generally closer to the null (data not shown).

To get a measure of potential impact, the proportional increase in IPTp delivery that would occur if selected barriers would have been removed or if drivers would have been fully scaled was estimated. Among the covariates that were found to be significant in the multivariable regression model, the following determinants were considered potentially modifiable: 1) removing user-fees for ANC medicines; 2) providing all facilities with IPTp guidelines/protocols; 3) implementing IPTp as part of the routine ANC services in all facilities; 4) preventing stock-outs of SP; and, 5) providing annual IPTp-specific training to ANC providers. As such, providers’ qualifications and client-level determinants such as education, age, weeks of pregnancy, or primigravidae status are not considered modifiable.

The modified PAF showed that, if the estimated effect size measures have a causal interpretation, removing user-fees for ANC medicines would have a negligible impact on delivery of IPTp (Table 2). This is not entirely surprising since the great majority of facilities in the five surveyed countries were not applying any charges. Similarly, providing IPTp guidelines/protocols to facilities would result in a 5.5% increase in IPTp delivery. Implementing routine IPTp in all facilities would increase by only 3.2% the delivery of IPTp during consultations, as 96% of facilities already offered IPTp as part of their ANC services. Preventing SP stock-outs would have a more important impact, increasing delivery of this intervention by 6.8%. These analyses suggest, however, that annual training of ANC providers for IPTp would have the biggest impact on IPTp delivery with an estimated 13.9% increase. Overall, addressing these five barriers/drivers jointly would result in a 30.6% (95% UI: 20.7-44.2%) increase in the proportion of client consultations where anti-malarial prophylaxis would have been administered as DOT.

**Table 2:** **Proportional increase attributable to selected modifiable determinants of intermittent preventive treatment for malaria in pregnancy delivery**

Barriers/Drivers	Current prevalence*	Proportional increase in IPTp (95% UI)
Removing user-fees for ANC medicines	12.2%	3.1% (1.4-6.5%)
Providing IPTp guidelines to all facilities	50.1%	5.5% (1.9-15.1%)
Integrating IPTp in routine ANC services	96.0%	3.2% (1.8-5.2%)
Preventing stock-outs of SP	10.7%	6.8% (4.1-10.4%)
Annual IPTp-training for providers	30.2%	13.9% (7.5-24.8%)
Joint effect		30.6% (20.7-44.2%)

UI = uncertainty intervals; SP = sulphadoxine-pyrimethamine; IPTp = intermittent preventive treatment for malaria in pregnancy; ANC = antenatal care.

*Prevalence estimates take into account the appropriate survey weights that were multiplied by the facility's number of ANC visits per month to represent the distribution of consultations for which the barrier/driver was present.

## Discussion

ANC attendance in sub-Saharan Africa has been referred to as a success story [39]. Among the five countries analysed in this paper, coverage for at least two ANC visits approximated 90% (Table 3). Coverage of IPTp, however, was much lower, varying between 11 and 27% according to demographic and health survey data. As current guidelines recommend a minimum of four antenatal visits, this IPTp gap represents considerable missed opportunities to deliver a cost-effective intervention. This paper has shown that the main potentially modifiable determinants of providers’ delivery of IPTp were the absence of user-fees for ANC medicines, availability of IPTp-specific guidelines/protocols in the facility, whether a facility claimed to routinely offer IPTp as part of their ANC services, the absence of SP stock-outs, and providers being recently trained for IPTp. The respective impact of these determinants on the expected proportional increase in IPTp delivery varied importantly, however. Training providers for IPTp would have the most impact with an expected 14% increase in delivery during ANC consultations. Addressing these five barriers/drivers jointly would increase the proportion of consultations where IPTp is delivered by 31%. These estimates depend strongly on the current distribution of these determinants, however, and countries where barriers are more common should expect higher impact (country-specific estimates can be found in Additional file 5).

**Table 3:** **Mothers’ attendance of antenatal care and uptake of IPTp in the five selected countries**

Country	Sample size*	≥1 ANC visit	≥2 ANC visits	≥1 dose of SP	≥2 doses of SP	Reference
Kenya	3,973 and 2,264	92.7%	88.4%	35.5%	15.1%	DHS 2008-09 [40]
Namibia	3,898 and 2,054	96.2%	94.5%	27.8%	10.6%	DHS 2006-07 [41]
Rwanda	3,658 and 2,267	97.3%	91.4%	53.0%	17.7%	DHS 2007-08 [42]
Tanzania	5,519 and 3,266	98.0%	94.4%	63.3%	27.2%	DHS 2010 [43]
Uganda	4,958 and 3,092	95.7%	91.7%	48.4%	26.7%	DHS 2011 [44]

ANC = antenatal care; SP = sulphadoxine-pyrimethamine; DHS = demographic and health survey.

*The first listed sample size corresponds to the denominator used to calculate ANC attendance and the second to the denominator use to estimate coverage of IPTp with SP. The sample sizes differ because the recall period for ANC attendance and IPTp coverage is for the most recent live birth over the last five years *versus* the last two years, respectively.

The fact that addressing these five determinants jointly would only lead to a 31% increase in IPTp delivery warrants further discussion as it emphasizes that no single determinant is responsible for the IPTp gap. Other factors, not measured in SPA surveys, could be important barriers to IPTp delivery. These include the unavailability of potable drinking water to swallow the SP tablets and/or sharing of drinking cups among clients [45, 46]; providers having high workload, low motivation, and/or being poorly organized [46–48]; providers’ confusion about timing and number of IPTp doses [11, 23]; women’s previous experiences of SP-related side effects and/or fear of side effects [49, 50]; women refusing to take the tablets on an empty stomach [12, 45]; and, women’s perception of malaria risk and their lack of knowledge about the benefits of IPTp [12, 51, 52]. Although the last three barriers do not correspond to supply-side determinants, providers could help mitigate/overcome them by providing women with appropriate information during their ANC consultation on the role, innocuity, and importance of IPTp.

Among the determinants that were not deemed to be modifiable, it was found that publicly managed facilities had higher rates of IPTp delivery than private ones. This could result from IPTp guidelines being easier to implement in public facilities as they are under the direct control of national authorities. The consequence being, as was observed in Nigeria, that providers from private facilities have incorrect knowledge of IPTp recommendations [53]. Further, local health authorities in Tanzania acknowledged that preferential treatment was provided to public facilities when it came to budget allocation for IPTp as they feared commercialization of this health service in private facilities [54].

The data showed that providers were 52% more likely to deliver IPTp if this was the client’s first ANC visit at the facility for their current pregnancy (conditional on being 16 weeks pregnant or more). Unclear policy and guidance for IPTp has been identified as a key barrier to effective delivery of this intervention and providers’ confusion about timing of the second SP dose could explain this result [12, 55]. In addition, providers were less likely to provide IPTp to women outside the 20^th^ to 32^nd^ weeks of pregnancy range. Tanzania and Namibia used an IPTp schedule that differed from WHO recommendations and this could have contributed to the observed pattern [56, 57] (i.e., administering IPTp between 20-24 and 28-32 weeks of gestation for Tanzania and between 26-28 and 34-36 weeks of gestation for Namibia).

Clinicians were the least likely to deliver IPTp to their clients. In ANC settings, these providers generally dispense palliative care for symptomatic pregnant women where IPTp might be contraindicated. Alternatively, it is also possible that nurses and midwives are more cognizant of the recommended standards of care and correctly apply the IPTp protocols. The fact that supervision of providers, a proxy for improved management practices, was not found to impact delivery of the intervention should not be taken at face value as the estimates could be affected by reverse causality. That is, underperforming providers that do not follow guidelines might attract supervisory attention.

This study has a number of limitations. First, several demand-side determinants of IPTp uptake were not collected by the SPA surveys. These include, for example, individual malaria risk perceptions and knowledge of benefits of anti-malarial prophylaxis during pregnancy. Although the omission of such important determinants contributes to increasing the standard errors of the estimates, they are likely not correlated to provider’s characteristics (conditional on the covariates included in the model) and should not bias the results. Second, information on the number and timing of the preceding dose(s) of SP, if any, was not available. The guidelines in effect at the time of the surveys recommended two doses of SP during the second and third trimester of pregnancy – with the exception of Kenya, which in 2006 recommended administration of IPTp at each scheduled ANC visits after quickening [58]. Hence, some of the clients could have been ineligible for IPTp if they had already received their two doses or if they had received their first one within the preceding four weeks. Coverage of two or more doses of IPTp is rather low in these countries (Table 3), however, and the likelihood that women were eligible for a third dose is small. Further, the four recommended ANC visits are usually scheduled at least one month apart, which would have also minimized the number of women ineligible for IPTp because their preceding dose was administered within the proscribed period. Another scenario for which women might have been ineligible for IPTp consists of SP being contra-indicated in HIV-infected women receiving co-trimoxazole (CTX) prophylaxis (but not those taking antiretroviral therapy). The proportion of women ineligible for IPTp based on CTX prophylaxis should be small, however, as implementation of this intervention has been slow in resource-limited settings [59]. Third, providers knew that they were being observed and the Hawthorn effect [60] could have affected the outcomes of ANC consultations. The most likely direction of this effect is that providers would have been more prone to administer anti-malarial prophylaxis, in which case the proportion of ANC consultations for which IPTp is given is probably lower than that reported in the surveys. However, if this effect is not correlated with the characteristics of the facility, the provider, or the client, then the effect size estimates should not be biased. Lastly, the proportional increase estimates derived from the PAF calculations assumes that the effect size measures have a causal interpretation. The lack of exogenous variation for the selected modifiable determinants prohibits such a strong causal claim. Yet, the estimates were robust to different model specifications and these determinants have been identified previously in both qualitative and quantitative studies [12, 15, 21, 23, 48, 53]. By combining information on exposure and effect size, it is believed that the PAF calculations shed an important light on the types of interventions most likely to increase coverage of IPTp.

This study is believed to be the first to use nationally representative survey data to assess determinants of provider’s delivery of IPTp during ANC consultations and to quantify the potential impact of these determinants. The use of standardized survey instruments enabled the pooling of data from surveys conducted in 1,285 facilities located in five countries. In addition, in comparison to other studies, an objectively measured outcome was used in lieu of women’s self-report of IPTp uptake (which is often affected by recall bias) [61].

## Conclusion

This study highlights a number of potential interventions to increase coverage of IPTp. User-fees for ANC medicines and facilities not having implemented routine IPTp are two important barriers to IPTp delivery, but these were relatively uncommon in the five surveyed countries. Close to half of facilities lack IPTp-specific guidelines/protocols but providing such items to all facilities would not increase delivery of this intervention substantially. In contrast, interventions aiming at improving the supply-chain management for SP to prevent stock-outs would have some impact, although modest, on delivery of IPTp. A quasi-experimental study conducted in the Zambia has shown that simple structural and information flow changes can lead to important improvements in the availability of drugs [62]. It is doubtful, however, that SP stock-outs could be entirely eliminated, especially in countries where parts of health budgets are contingent upon external funding [63]. As such, the proportional increase in IPTp delivery that was estimated should be interpreted as a theoretical upper bound. One of the most interesting findings of this study was that training of providers could lead to important increases in IPTp delivery. Only 30% of ANC consultations were offered by providers who had received IPTp training during the year before the survey, leaving room for improvements.

Interventions aimed at increasing knowledge and competency of health workers are available and have shown positive impacts on delivery of IPTp in Uganda [21] and Kenya [23]. Future studies should evaluate the potential of targeting interventions, either spatially in areas of low IPTp coverage or to specific facilities. Private facilities, health post and dispensary were shown to be less likely to administer IPTp and should be prioritized to increase coverage of this intervention. Multi-pronged approaches are most likely to yield substantial increases in IPTp coverage, and addressing providers’ barriers to effective IPTp delivery is urgently required if the Roll Back Malaria Partnership goal of 100% coverage by 2015 [13] and the President’s Malaria Initiative target of 85% coverage [14] are to be achieved.

## Electronic supplementary material

Additional file 1: **Harmonization of the classification schemes for type of facility and the provider’s qualification.** This table provides information on the different classification schemes for facility types and provider’s qualifications were harmonized to be used in the pooled analysis. (PDF 68 KB)

Additional file 2: **Country-specific multivariable results of the modified Poisson regression models of providers’ determinants of delivery of intermittent preventive treatment for malaria in pregnancy administered as directly observed therapy.** Presented in this table are the multivariable results when the regression models were run separately for Kenya, Namibia, Rwanda, Tanzania, and Uganda. (PDF 158 KB)

Additional file 3: **Multivariable results of the modified Poisson regression models, with fixed effect at the regional level, of providers’ determinants of delivery of intermittent preventive treatment for malaria in pregnancy (IPTp) administered as directly observed therapy.** This regression model controls for 60 dummy variables that correspond to the different regions of the five surveyed countries. Controlling for these regional variables had no effect on the main determinants of IPTp delivery. (PDF 89 KB)

Additional file 4: **Multivariable results of the modified Poisson regression models of providers’ determinants of delivery of intermittent preventive treatment for malaria in pregnancy administered as directly observed therapy (IPTp) in facilities that claim that this intervention is routinely offered as part of their antenatal care services (ANC).** Facilities that do not claim that IPTp is part of their routine ANC services were excluded. Results show that this has no impact on the main determinants of IPTp delivery. (PDF 90 KB)

Additional file 5: **Proportional increase attributable to selected modifiable determinants of intermittent preventive treatment for malaria in pregnancy (IPTp, administered as directly observed therapy) for Kenya, Namibia, Rwanda, Tanzania, and Uganda.** The proportional increase in IPTp delivery was calculated using the population attributable fraction for each country separately. (PDF 63 KB)
