# Supplementary material for: Factors affecting providers’ delivery of intermittent preventive treatment for malaria in pregnancy: a five-country analysis of national service provision assessment surveys
Source: Malar J. 2014 Nov 20;13:440. doi: 10.1186/1475-2875-13-440 (PMC4247687; doi:10.1186/1475-2875-13-440)
Supplement: Supplementary file 1 — Additional file 1: Harmonization of the classification schemes for type of facility and the provider’s qualification. This table provides information on the different classification schemes for facility types and provider’s qualifications were harmonized to be used in the pooled analysis. (PDF 68 KB) [file 12936_2014_3599_MOESM1_ESM.pdf]

ADDITIONAL FILE 1

Harmonization of the classification schemes for type of facility and the provider's qualification

	Adopted classification	Service provision assessment surveys<br>Kenya 2010	Namibia 2009	Rwanda 2007	Tanzania 2006	Uganda 2007
Facility type	Health centre	Health centre<br>Maternity	Health centre	Health centre<br>Polyclinic	Health centre	Health centre IV
	Health post/ Dispensary	Clinic<br>Dispensary	Clinic	Dispensary<br>Health post<br>Clinic	Dispensary<br>Stand-alone	Health centre II<br>Health centre III
	Hospital	National hospital<br>Provincial hospital<br>District hospital<br>Sub-district hospital<br>Other hospital	Hospital	Hospital	Hospital	Hospital
Provider's qualification	Clinicians	Specialist<br>Medical officer<br>Clinical officer	Medical officer<br>Physician	Gynaeco-obstetrician	Consultant<br>Medical doctor<br>Medical officer<br>Clinical officer	Consultant<br>Medical doctor<br>Clinical officer
	Registered nurse/Midwife	Registered nurse<br>Registered midwife	Registered nurse<br>Registered midwife	Nurse A2	Registered nurse<br>Nursing officer<br>Public health nurse	Registered nurse<br>Registered midwife<br>Comprehensive nurse<br>Public health nurse
	Enrolled nurse/ Midwife	Enrolled nurse<br>Enrolled midwife	Enrolled nurse<br>Enrolled midwife	Nurse A3	Nurse midwife<br>Trained nurse	Enrolled nurse<br>Enrolled midwife
	Other	BSN nurse<br>Nurse aide<br>Nutritionist<br>Other staff	Medical assistant<br>Nurse assistant	Health auxiliary<br>Social assistant A2<br>Other	Clinical assistant<br>Laboratory assistant<br>Auxiliary nurse<br>Medical assistant	Nursing assistant<br>Nursing aide
