# Supplementary material for: Factors affecting providers’ delivery of intermittent preventive treatment for malaria in pregnancy: a five-country analysis of national service provision assessment surveys
Source: Malar J. 2014 Nov 20;13:440. doi: 10.1186/1475-2875-13-440 (PMC4247687; doi:10.1186/1475-2875-13-440)
Supplement: Supplementary file 2 — Additional file 2: Country-specific multivariable results of the modified Poisson regression models of providers’ determinants of delivery of intermittent preventive treatment for malaria in pregnancy administered as directly observed therapy. Presented in this table are the multivariable results when the regression models were run separately for Kenya, Namibia, Rwanda, Tanzania, and Uganda. (PDF 158 KB) [file 12936_2014_3599_MOESM2_ESM.pdf]

ADDITIONAL FILE 2

Country-specific multivariable results of the modified Poisson regression models of providers' determinants of delivery of intermittent preventive treatment for malaria in pregnancy administered as directly observed therapy

**Variables**	**Kenya 2010 RR (95% CI)**	**Namibia 2009 RR (95% CI)**	**Rwanda 2007 RR (95% CI)**	**Tanzania 2006 RR (95% CI)**	**Uganda 2007 RR (95% CI)**
	N=1,303	N=610	N=660	N=1,521	N=728
**Facilities**					
Facility type (Health centre)	Reference	Reference	Reference	Reference	Reference
Hospital	1.03 (0.84-1.27)	-*	-*	0.89 (0.59-1.32)	0.99 (0.75-1.32)
Health post/Dispensary	0.95 (0.71-1.26)	0.76 (0.37-1.59)	0.97 (0.73-1.29)	0.77 (0.53-1.13)	**0.58 (0.40-0.86)**
Public facility	**1.81 (1.34-2.45)**	-*	1.07 (0.87-1.33)	1.39 (0.97-1.98)	1.06 (0.80-1.40)
Facility has fee for ANC medicines	0.93 (0.75-1.15)	-*	0.28 (0.06-1.37)	0.40 (0.13-1.26)	-*
Facility has IPTp guidelines	**1.25 (1.04-1.50)**	4.06 (0.43-38.27)	0.85 (0.71-1.02)	1.21 (0.95-1.55)	1.06 (0.83-1.36)
Facility claims routine IPTp	3.54 (0.52-24.03)	3.31 (0.89-12.29)	-*	5.67 (0.92-34.85)	-*
SP stocked-out on visit day	0.66 (0.36-1.20)	0.16 (0.03-1.05)	**0.27 (0.09-0.83)**	**0.31 (0.16-0.59)**	**0.09 (0.02-0.42)**
**Providers**					
Provider is a physician	Reference	-*	-*	Reference	-*
Enrolled nurse/Midwife	**4.72 (1.18-18.87)**	Reference	Reference	1.86 (0.80-4.32)	Reference
Registered nurse/Midwife	**4.88 (1.23-19.47)**	1.00 (0.54-1.85)	0.98 (0.67-1.44)	1.71 (0.76-3.84)	1.07 (0.84-1.36)
Other	2.96 (0.48-18.23)	-*	0.99 (0.66-1.51)	1.96 (0.89-4.33)	0.95 (0.56-1.61)
Supervised in last 6 months	**0.76 (0.62-0.93)**	0.68 (0.37-1.24)	1.05 (0.77-1.42)	**1.45 (1.02-2.06)**	1.29 (0.84-1.97)
Trained for IPTp in last year	1.19 (1.00-1.42)	1.47 (0.70-3.08)	1.07 (0.89-1.28)	1.25 (0.95-1.63)	1.25 (0.98-1.61)
**Consultations/Clients**					
Primigravidae	1.06 (0.93-1.20)	0.94 (0.51-1.76)	1.00 (0.87-1.15)	1.11 (0.90-1.37)	1.06 (0.89-1.25)
First visit at facility	**1.53 (1.33-1.75)**	1.50 (0.75-3.00)	1.17 (0.99-1.38)	**1.51 (1.26-1.81)**	**1.75 (1.38-2.22)**
Education (None)	Reference	Reference	Reference	Reference	Reference
Primary	1.14 (0.92-1.41)	1.54 (0.59-4.06)	0.90 (0.80-1.00)	0.98 (0.83-1.16)	1.01 (0.83-1.22)
Secondary/Higher	1.20 (0.95-1.50)	2.01 (0.81-4.96)	0.80 (0.63-1.03)	0.84 (0.61-1.15)	1.04 (0.84-1.29)
Age (<20 years)	Reference	Reference	Reference	Reference	Reference
20-29 years	0.97 (0.84-1.13)	0.77 (0.45-1.31)	0.90 (0.71-1.15)	1.22 (0.94-1.59)	1.09 (0.88-1.35)
≥30 years	1.04 (0.87-1.24)	**0.19 (0.07-0.54)**	0.87 (0.66-1.15)	1.00 (0.75-1.35)	1.03 (0.81-1.31)
Cubic b-spline for weeks of pregnancy (5 degrees of freedom; the grey area represents the 95% confidence intervals)	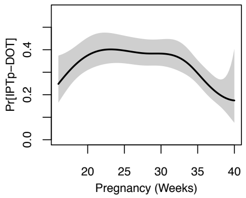	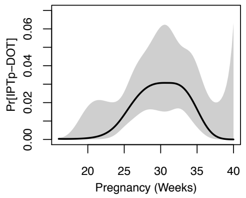	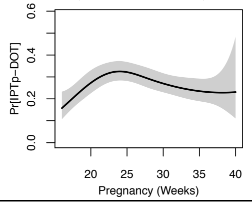	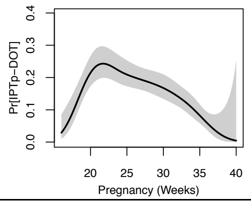	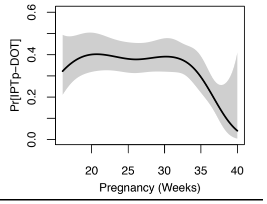

Statistically significant results at the α=0.05 level are bolded
RR (95% CI) = relative risk with 95% confidence intervals; IPTp = intermittent preventive treatment for malaria in pregnancy; ANC = antenatal care.
Total number of observations does not match the one from the combined analyses because some categories of variables coded with a missing indicators could not be estimated and these observations had to be excluded from the analyses to ensure numerical stability.
-* Not enough within-country variation for the effect size of the covariate to be estimated.
