# Supplementary material for: Factors affecting providers’ delivery of intermittent preventive treatment for malaria in pregnancy: a five-country analysis of national service provision assessment surveys
Source: Malar J. 2014 Nov 20;13:440. doi: 10.1186/1475-2875-13-440 (PMC4247687; doi:10.1186/1475-2875-13-440)
Supplement: Supplementary file 3 — Additional file 3: Multivariable results of the modified Poisson regression models, with fixed effect at the regional level, of providers’ determinants of delivery of intermittent preventive treatment for malaria in pregnancy (IPTp) administered as directly observed therapy. This regression model controls for 60 dummy variables that correspond to the different regions of the five surveyed countries. Controlling for these regional variables had no effect on the main determinants of IPTp delivery. (PDF 89 KB) [file 12936_2014_3599_MOESM3_ESM.pdf]

ADDITIONAL FILE 3

Multivariable results of the modified Poisson regression models, with fixed effect at the regional level, of providers' determinants of delivery of intermittent preventive treatment for malaria in pregnancy administered as directly observed therapy

Variables	IPTp administered as DOT RR (95% CI)
	**N=4,971**
**Fixed effects for regions**	60 Dummies
**Facilities**	
Facility type (Health centre)	Reference
Hospital	1.00 (0.87-1.16)
Health post/Dispensary	**0.84 (0.71-0.99)**
Public facility	**1.22 (1.06-1.40)**
Facility has fee for ANC medicines	**0.75 (0.61-0.92)**
Facility has IPTp guidelines	1.10 (0.99-1.22)
Facility claims routine IPTp	**3.21 (1.36-7.56)**
SP stocked-out on visit day	**0.42 (0.29-0.62)**
**Providers**	
Provider is a physician	Reference
Enrolled nurse/Midwife	**3.96 (1.86-8.40)**
Registered nurse/Midwife	**3.92 (1.85-8.32)**
Other	**4.17 (1.94-8.97)**
Supervised in last 6 months	0.99 (0.86-1.14)
Trained for IPTp in last year	**1.14 (1.03-1.26)**
**Consultations/Clients**	
Primigravidae	1.01 (0.94-1.10)
First visit at facility	**1.50 (1.38-1.64)**
Education (None)	Reference
Primary	0.98 (0.90-1.06)
Secondary/Higher	1.02 (0.92-1.13)
Age (<20 years)	Reference
20-29 years	0.97 (0.87-1.08)
≥30 years	0.90 (0.80-1.02)
Cubic b-spline for weeks of pregnancy (5 degrees of freedom; the grey area represents the 95% confidence intervals)	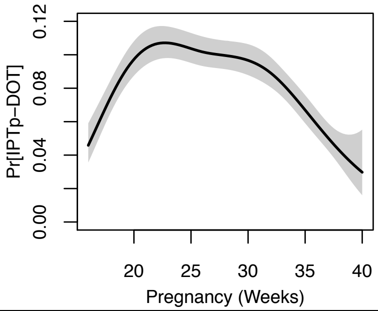

Statistically significant results at the α=0.05 level are bolded

RR (95% CI) = relative risk with 95% confidence intervals; SP = sulphadoxine-pyrimethamine; IPTp = intermittent preventive treatment for malaria in pregnancy; DOT = directly observed therapy; ANC = antenatal care.
