# Supplementary material for: Factors affecting providers’ delivery of intermittent preventive treatment for malaria in pregnancy: a five-country analysis of national service provision assessment surveys
Source: Malar J. 2014 Nov 20;13:440. doi: 10.1186/1475-2875-13-440 (PMC4247687; doi:10.1186/1475-2875-13-440)
Supplement: Supplementary file 4 — Additional file 4: Multivariable results of the modified Poisson regression models of providers’ determinants of delivery of intermittent preventive treatment for malaria in pregnancy administered as directly observed therapy (IPTp) in facilities that claim that this intervention is routinely offered as part of their antenatal care services (ANC). Facilities that do not claim that IPTp is part of their routine ANC services were excluded. Results show that this has no impact on the main determinants of IPTp delivery. (PDF 90 KB) [file 12936_2014_3599_MOESM4_ESM.pdf]

ADDITIONAL FILE 4

Table 4 Multivariable results of the modified Poisson regression models of providers' determinants of delivery of intermittent preventive therapy for malaria in pregnancy (administered as directly observed therapy) in facilities that claim that this intervention is routinely offered as part of their antenatal care services

Variables	IPTp administered as DOT<br>RR (95% CI)
	**N=4,668**
**Country** (Kenya 2010)	Reference
Namibia 2009	**0.23 (0.16-0.33)**
Rwanda 2007	1.16 (0.97-1.39)
Tanzania 2006	**0.67 (0.56-0.80)**
Uganda 2007	**0.83 (0.71-0.97)**
**Facilities**	
Facility type (Health centre)	Reference
Hospital	1.03 (0.88-1.19)
Health post/Dispensary	**0.82 (0.69-0.98)**
Public facility	**1.22 (1.06-1.41)**
Facility has fee for ANC medicines	**0.75 (0.61-0.92)**
Facility has IPTp guidelines	**1.12 (1.00-1.24)**
SP stocked-out on visit day	**0.41 (0.28-0.61)**
**Providers**	
Provider is a physician	Reference
Enrolled nurse/Midwife	**3.46 (1.54-7.79)**
Registered nurse/Midwife	**3.53 (1.57-7.95)**
Other	**3.77 (1.65-8.60)**
Supervised in last 6 months	0.99 (0.85-1.14)
Trained for IPTp in last year	**1.21 (1.09-1.35)**
**Consultations/Clients**	
Primigravidae	1.02 (0.94-1.10)
First visit at facility	**1.53 (1.40-1.67)**
Education (None)	Reference
Primary	0.98 (0.90-1.06)
Secondary/Higher	1.03 (0.93-1.14)
Age (<20 years)	Reference
20-29 years	0.99 (0.90-1.10)
≥30 years	0.93 (0.82-1.05)
Cubic b-spline for weeks of pregnancy (5 degrees of freedom; the grey area represents the 95% confidence intervals)	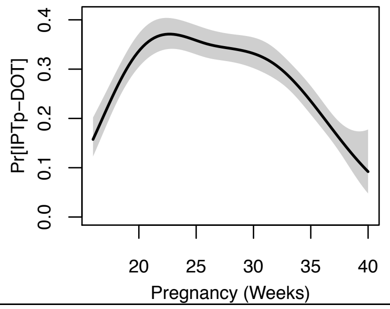

Statistically significant results at the α=0.05 level are bolded; RR (95% CI) = relative risk with 95% confidence intervals; SP = sulphadoxine-pyrimethamine; IPTp = intermittent preventive treatment for malaria in pregnancy; DOT = directly observed therapy; ANC = antenatal care.
