# Supplementary material for: Factors affecting providers’ delivery of intermittent preventive treatment for malaria in pregnancy: a five-country analysis of national service provision assessment surveys
Source: Malar J. 2014 Nov 20;13:440. doi: 10.1186/1475-2875-13-440 (PMC4247687; doi:10.1186/1475-2875-13-440)
Supplement: Supplementary file 5 — Additional file 5: Proportional increase attributable to selected modifiable determinants of intermittent preventive treatment for malaria in pregnancy (IPTp, administered as directly observed therapy) for Kenya, Namibia, Rwanda, Tanzania, and Uganda. The proportional increase in IPTp delivery was calculated using the population attributable fraction for each country separately. (PDF 63 KB) [file 12936_2014_3599_MOESM5_ESM.pdf]

ADDITIONAL FILE 5

Table 5 Proportional increase attributable to selected modifiable determinants for intermittent preventive treatment for malaria in pregnancy (administered as directly observed therapy) for Kenya, Namibia, Rwanda, Tanzania, and Uganda.

Barriers/Drivers	Current prevalence*	Proportional increase in IPTp (95% UI) †
**Kenya**		
Removing user-fees for ANC medicines	30.6%	8.0% (3.6-17.4%)
Providing IPTp guidelines to all facilities	45.1%	6.1% (2.1-16.7%)
Integrating IPTp in routine ANC services	94.5%	4.4% (1.6-8.0%)
Preventing stock-outs of SP	11.1%	7.1% (3.0-12.1%)
Annual IPTp-training for providers	28.4%	14.3% (7.6-25.6%)
Joint effect		40.4% (26.5-58.7%)
**Namibia**		
Removing user-fees for ANC medicines	0.4%	0.1% (0.1-0.3%)
Providing IPTp guidelines to all facilities	0.4%	11.7% (3.6-38.0%)
Integrating IPTp in routine ANC services	57.1%	48.4% (14.1-95.7%)
Preventing stock-outs of SP	14.9%	9.8% (3.4-17.5%)
Annual IPTp-training for providers	22.9%	15.6% (7.9-28.9%)
Joint effect		80.3% (33.7-130.9%)
**Rwanda**		
Removing user-fees for ANC medicines	5.1%	1.3% (0.2-2.9%)
Providing IPTp guidelines to all facilities	53.2%	5.2% (1.7-14.4%)
Integrating IPTp in routine ANC services	97.1%	2.2% (0.3-4.7%)
Preventing stock-outs of SP	9.3%	5.9% (2.4-10.0%)
Annual IPTp-training for providers	24.0%	15.3% (8.2-27.8%)
Joint effect		31.4% (20.2-46.4%)
**Tanzania**		
Removing user-fees for ANC medicines	4.6%	1.1% (0.3-2.5%)
Providing IPTp guidelines to all facilities	53.3%	5.2% (1.8-14.0%)
Integrating IPTp in routine ANC services	95.4%	3.6% (1.4-6.5%)
Preventing stock-outs of SP	14.7%	9.7% (5.4-15.2%)
Annual IPTp-training for providers	17.5%	16.8% (9.0-30.9%)
Joint effect		40.0% (25.8-58.0%)
**Uganda**		
Removing user-fees for ANC medicines	4.1%	1.0% (0.1-2.4%)
Providing IPTp guidelines to all facilities	51.3%	5.4% (1.7-14.8%)
Integrating IPTp in routine ANC services	99.6%	0.3% (0.1-0.9%)
Preventing stock-outs of SP	7.2%	4.5% (0.6-9.1%)
Annual IPTp-training for providers	40.3%	11.6% (5.8-21.5%)
Joint effect		20.1% (11.4-31.9%)

UI = uncertainty intervals; SP = sulphadoxine-pyrimethamine; IPTp = intermittent preventive treatment for malaria in pregnancy; ANC = antenatal care

* Prevalence estimates were weighted using the appropriate survey weights multiplied by the number of ANC visits per month at the facility to represent the distribution of consultations for which the barrier/driver was present.

† Relative risks from the pooled model were used.
